# Gel-Based Materials for Ophthalmic Drug Delivery

**DOI:** 10.3390/gels7030130

**Published:** 2021-08-29

**Authors:** Roberta Cassano, Maria Luisa Di Gioia, Sonia Trombino

**Affiliations:** Department of Pharmacy, Health and Nutritional Sciences, University of Calabria, Arcavacata di Rende, 87036 Cosenza, Italy; Roberta.cassano@unical.it (R.C.); ml.digioia@unical.it (M.L.D.G.)

**Keywords:** gels, hydrogels, gel eye drops, in situ gels, intravitreal injection, contact lens

## Abstract

The most common route of administration of ophthalmic drugs is the topical route because it is convenient, non-invasive, and accessible to all patients. Unfortunately, drugs administered topically are not able to reach effective concentrations. Moreover, their bioavailability must be improved to decrease the frequency of administrations and their side effects, and to increase their therapeutic efficiency. For this purpose, in recent decades, particular attention has been given to the possibility of developing prolonged-release forms that are able to increase the precorneal residence time and decrease the loss of the drug due to tearing. Among these forms, gel-based materials have been studied as an ideal delivery system because they are an extremely versatile class with numerous prospective applications in ophthalmology. These materials are used in gel eye drops, in situ gelling formulations, intravitreal injections, and therapeutic contact lenses. This review is intended to describe gel-based materials and their main applications in ophthalmology.

## 1. Introduction

**Figure 1:**
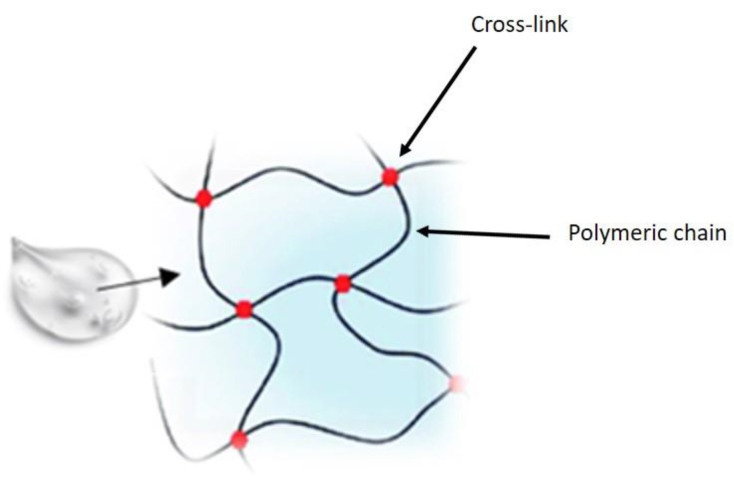
Hydrogel structure.

Eye drops are the preferred method of drug administration for many eye diseases due to their convenience and relatively low cost. Unfortunately, their use is strongly conditioned by the difficult penetration of the drugs administered into the internal structures of the eye itself, represented by the epithelia of the cornea and conjunctiva, and this leads to poor ocular bioavailability (<5%) [1]. Therefore, to obtain a therapeutic efficacy, it is required to reach high concentrations in the ocular tissues. This can cause side effects such as toxicity and low tolerability, which are often associated with poor compliance by the patient, an important limiting factor for many topical ophthalmic medications [2]. In recent years, pharmaceutical research has focused attention on the development of new drug delivery approaches aimed at increasing ocular residence time, providing prolonged pharmacological action, and improving bioavailability, thus minimizing side effects and improving patient safety. The various strategies employed to increase the residence time of drugs at the precorneal level and, therefore, their bioavailability, include the use of gels, which are materials composed of a three-dimensional cross-linked polymer or a colloidal network immersed in a fluid. Some of these, defined hydrogels, have water as their main constituent and are widely used in the ophthalmic field. Hydrogels comprise three-dimensional, hydrophilic, polymeric networks capable of absorbing large amount of water or biological fluids, due to the presence of hydrophilic groups, and releasing the drugs entrapped in them through slow diffusion [3] (Figure 1).

Hydrogels enable the incorporation of a variety of ophthalmic pharmaceuticals, are retained in the eye, and are well tolerated compared to conventional pharmaceutical formulations such as ointments, while simultaneously decreasing the side effects due to systemic absorption [4].

Ophthalmic gels are divided into two categories: gel eye drops and in situ gels [5]. The first exist as viscous solutions before application to the eye and are normally used for dry eyes as a tear substitute [6]. In situ gels, by comparison, are liquids that are applied as drops onto the eye and only after administration undergo a sol-gel-to-gel transition in the conjunctival cul-de-sac following external stimuli, such as pH, temperature, or ions, with a significant improvement in ocular bioavailability [7]. Furthermore, hydrogels are also applied in intravitreal injections or used in the form of contact lenses [8].

In this context, this review intends to focus attention on the gel-based materials used in the treatment of eye diseases.

## 2. Ophthalmic Gels

### 2.1. Gel Eye Drops

**Figure 2:**
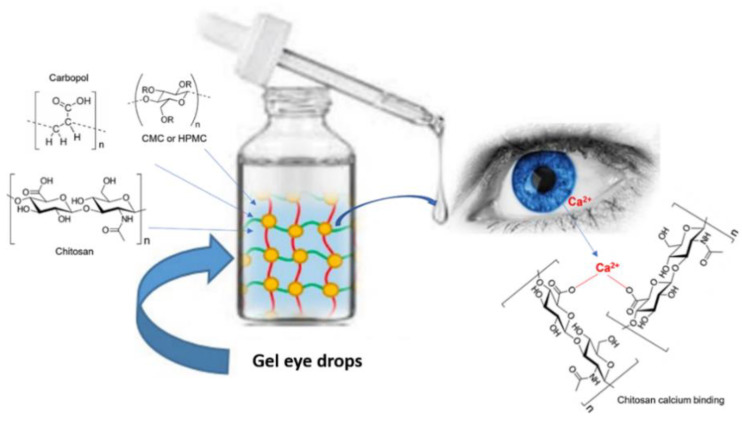
Administration of gel eye drops to the eye.

Gel eye drops are simple viscous formulations that do not undergo any changes after their administration (Figure 2). They are mainly applied as tear substitutes in the treatment of dry eye because they present numerous drawbacks, which limit their use for the administration of ophthalmic drugs. Gel eye drops do not allow accurate and reproducible drug administration and can cause blurred vision, crusting on the eyelids, and tearing [9,10,11].

In general, these formulations consist of cellulose derivatives, such as carboxymethylcellulose (CMC) [12,13], hydroxypropylmethylcellulose (HPMC) [14,15], carbomer (carbopol), or hyaluronic acid (HA) [16,17,18]. Cellulose derivatives were the first polymeric materials to be applied as excipients in ophthalmic pharmaceutical forms [19]. In eye drops, HPMC is used to increase viscosity, mucoadhesion, and stability of the formulation [14,15,19,20,21]. By comparison, hyaluronic acid (HA), and alginic acid (ALG) are sometimes preferred to cellulosic derivatives for specific applications. Hyaluronic acid is used as an excipient in artificial tears, due to both its protective effect against the damage caused by benzalkonium chloride, a preservative commonly used in eye drops [22], and to its pseudoplastic behavior that ensures good diffusion of the polymer on the ocular surface at the moment of blinking [18].

Another important polymer chosen to overcome the rapid elimination of instilled ophthalmic solutions is chitosan [23]. Formulations based on chitosan appear to be less viscous than those based on HA. Additionally, chitosan has the advantage of possessing positive charges on its skeleton which, at the physiological pH level, interact with the negative charges of the ocular mucus, thus improving bioadhesion. [23,24,25]. In addition, the polysaccharide alginic acid is applied in ophthalmic formulations because its solutions can be cross-linked to form a hydrogel by a mild gelation reaction following exposure to Ca^2+^ ions [19].

Carbopol is one of the most used polymers in ophthalmic formulations. It is a cross-linked homopolymer of acrylic acid which has good bioadhesive properties. Furthermore, at physiological pH, it can interact with mucus and biological surfaces through the hydrogen bonding of the ionized carbonyl functionalities [26] with the formation of a reinforced gel lattice that allows the particles to remain adhesive for long periods of time. Many commercial eye drops contain carbopol 940 to achieve better corneal retention and greater bioavailability. In this regard, Mona et al. [27] designed and manufactured gel-core liposomes as advanced systems for improving ocular drug delivery and residence time. Fluconazole (FLZ) was chosen because it is one of the main ocular antifungals characterized by poor corneal permeation and short residence time in the precorneal area. Therefore, gel-core carbosomes were developed as novel ophthalmic carbopol-based vehicles, specifically to address the obstacles related to the ocular administration of FLZ and support its effect. Furthermore, the important role of gel-core carbosomes as a vehicle for FLZ was demonstrated in ex vivo and in vivo corneal permeation studies, in which it was shown that FLZ deposition increased and, simultaneously, gel-core carbosomes were also able to protect FLZ from degradation and metabolism, thus improving ocular activity and therapeutic effect [27].

### 2.2. In Situ Gels

**Figure 3:**
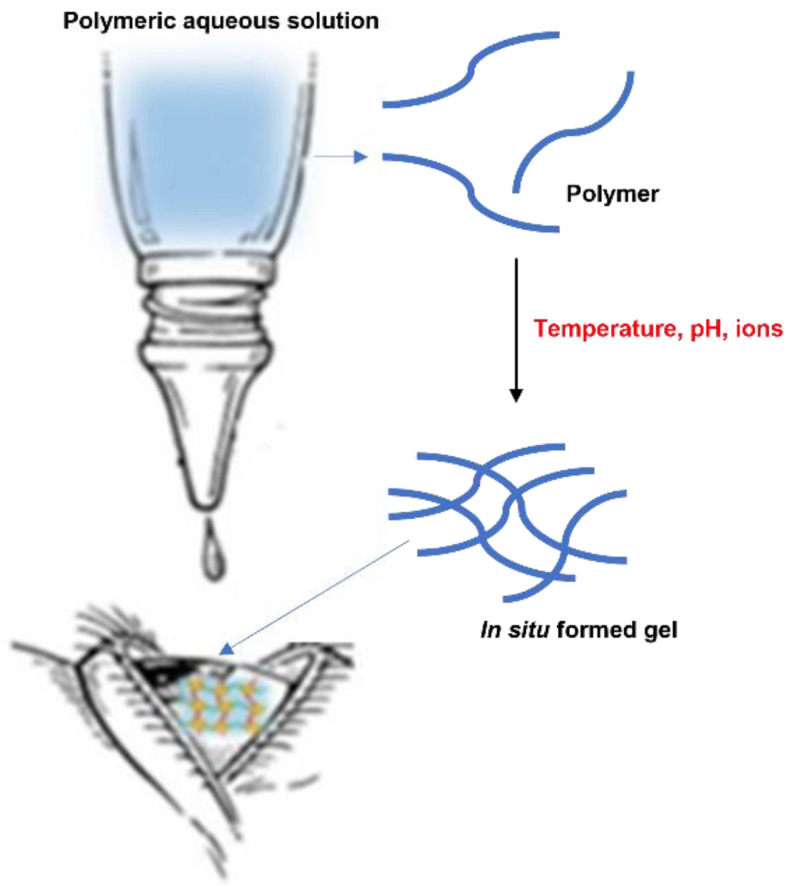
In situ gelling system.

The in situ gelling system represents one of the most interesting strategies and a promising approach to increase the residence time of drugs on the ocular surface (Figure 3).

After instillation of the aqueous solution containing polymers sensitive to external stimuli, a viscous and mucoadhesive gel is formed on the surface of the eye [28], with a consequent increase in the ocular retention time and bioavailability of the drugs administered. Furthermore, in situ gelling system are characterized by a number of advantages, such as the simple manufacturing process, the ease of administration, and the delivery of an accurate dose [29].

Gelation can be caused by various external stimuli, such as temperature, pH, and ions. The in situ activated temperature gelling system is based on the use of polymers that are in liquid form below their low critical solution temperature (LCST) and become gels when the ambient temperature is equal to or above the LCST [30].

pH-induced in situ gels consist of polymers that possess acidic or alkaline functional groups. They gel during the transition from a low pH environment to a high pH environment. Furthermore, in ionic systems the polymer undergoes a sol-gel transition due to changes in ionic concentration, which are typically triggered by mono or divalent cations present in tear fluid, such as Na^+^, Mg^2+^, and Ca^2+^ ions [31].

#### 2.2.1. Temperature-Sensitive In Situ Gel Systems

This type of formulation can be applied to the eye in liquid form and the gel is formed at the precorneal temperature of 35 °C [32]. It is important that this type of gel has a gelation temperature higher than room temperature and undergoes a gel-sol transition at the precorneal temperature. Thus, it is recommended to avoid storing the formulation in the refrigerator before instillation, because this could cause eye irritation due to its cold temperature [33].

In situ heat-sensitive gelling formulations of antifungal ketoconazole (KCL) based on poly (N-isopropylacrylamide)/hyaluronic acid (PN-HA) have been prepared and characterized for in vitro gelation, drug release, and antifungal activity [34]. The content of the drug in the prepared gels was found to be between 91% and 96%. The pH value was between 6.0 and 7.5 and, therefore, compatible with the eye. The gelation temperature of the prepared PN-HA based solutions was found to be 33 °C. The release of KCL from the gels in situ was moderate and no bursting effects occurred. Additionally, these gels were well tolerated by rabbits without causing irritation, redness, or other toxic effects. Furthermore, in vivo antimicrobial tests revealed that PN-HA gels for in situ delivery of KCL can accelerate the healing process by slowing the growth of *Candida albicans*. Therefore, this new formulation may represent an interesting dosage form capable of prolonging residence time and controlling the release of KCL into the eye [34].

Okur et al. developed new ocular in situ gel formulations containing voriconazole, (VCZ) a drug useful for the treatment of fungal keratitis, and evaluated them as an effective means of topical ocular delivery [35]. For this reason, in situ eye gels were subjected to physico-chemical, rheological characterization. Stability, in vitro release, microbiological tests, and ex vivo and in vivo evaluations were also performed using New Zealand albino rabbits. In situ gel formulations triggered by temperature were obtained via the cold method. Poloxamer 188, poloxamer 407, and carboxymethylcellulose were used for the preparation of gels. The gelation temperatures of the formulations were in the range of 29–34 °C. The drug release results indicated that all formulations were successful in ensuring sustained release of voriconazole and were found to be stable for 3 months. Following the administration of the gels, irritation tests were performed that did not reveal either eye damage or clinically abnormal signs in the cornea, conjunctiva, or iris. Therefore, such gels could represent promising ocular vectors for the administration of voriconazole in the treatment of fungal keratitis. [35].

Recently, Gugleva obtained an in situ heat-sensitive niosomal gel of antibiotic doxycycline for ophthalmic application [36]. For this purpose, in situ gel formulations based on poloxamer 407 alone and in combination with HPMC were prepared and evaluated in terms of sol-gel transition temperature, gelation time, and capacity. The addition of HPMC to the formulation caused a decrease in the phase transition temperature of the systems, whereas the inclusion of doxycycline niosomes in the formulations did not significantly influence their gelling properties or their rheological properties. Poloxamer- and HPMC-based niosomal doxycycline gels in situ exhibited a gelation temperature of 34 °C, a pseudoplastic flow behavior, and physical stability, and were therefore found to be suitable for ophthalmic applications. The results obtained indicated that the niosomal gel in situ can act as a promising system for the ophthalmic administration of doxycycline, ensuring a sufficient therapeutic concentration and a prolonged release of the drug.

Wei and collaborators developed and characterized two heat-sensitive in situ gels based on poloxamer 407 and methylcellulose, and used betaxol, a beta-adrenergic receptor inhibitor indicated for the treatment of chronic open-angle glaucoma or ocular hypertension, as a model drug [37]. Hence, the aim of their research was to analyze the effect of drug release in relation to the type of gel and the gelation temperature. It was also investigated whether the in situ gel drug delivery system possessed comparable in vivo behavior to that of the commercial resin suspension. In particular, in vitro tests were conducted to evaluate the release of betaxol and in vivo pharmacokinetic studies were aimed at evaluating the distribution of the drug in the ocular tissue.

Furthermore, in situ gels were investigated and compared with betaxol (Betoptic S^®^ China Pioneer Pharma Holdings Limited, Shanghai) eye drops, commercially available suspension eye drops, using ion exchange (IR) resins to support drug release, approved by the FDA. In vivo pharmacokinetic data indicated that the in situ gels were superior in increasing the drug concentration in the cornea. Therefore, they were applied to those drugs that are not suitable for ion exchange resins and, consequently, are a promising delivery system for prolonged administration of ophthalmic drugs with wide drug adoptability.

Another interesting study was performed by Mahboobian and coworkers, in which thermosensitive in situ gel nanoemulsions (NEs) containing acyclovir (ACV) for ocular drug delivery were formulated and their ex vivo corneal permeation ability was evaluated [38]. The NEs loaded with ACV were prepared by the low energy method. The optimal NE gel in situ was made using Triacetin, Transcutol P^®^ ((diethylenglycol monoethyl ether) were kindly supplied by Gattefossé, St. Priest, France), poloxamer 407, and poloxamer 188, which showed an average droplet size of 28 nm and a polydispersion index of 0.38. Evaluation tests on physico-chemical properties revealed that the formulation obtained was suitable for ocular administration. Additionally, permeation studies indicated that the amount of drug released from the formulation was approximately 2.8-fold greater than that of the control solution. Irritation tests also confirmed that the optimal NE gel in situ, being well tolerated by the eye, may be considered to be a valid delivery system of ophthalmic drugs in the treatment of viral ocular infections [38].

In 2020, Kim and collaborators proposed and described a hypotonic gelling solution containing a low concentration of a particular three-block thermosensitive copolymer for prolonged administration of the ocular drug. In particular, following topical application, the hypotonic formulation formed a highly uniform and transparent thin layer that adapted to the ocular surface and even resisted the elimination of the formulation due to blinking. This causes an increase in intraocular absorption of both hydrophilic and hydrophobic drugs, thus extending the drug-ocular epithelium contact time compared to conventional heat-sensitive gelling formulations and commercial eye drops [39].

#### 2.2.2. pH-Sensitive In Situ Gel Systems

In situ gels can be formed because of changes in pH. At the value of 4.4, they occur in the form of solutions that undergo coagulation when the pH rises. After contact with the tear fluid, a viscous gel is formed. These polymers contain acidic or basic groups that accept or release protons in response to changes in environmental pH, and then swell, thus releasing the contained drug. The swelling of the hydrogel increases with increasing external pH in the case of weakly acidic (anionic) groups. Most pH-sensitive anionic polymers are based on carbopol, carbomer, or its derivatives, but those based on polyacrylic acid (PAA) are also effective. PAA solutions gel at pH 7.4 and high concentrations and this, given the low pH of the PAA solution, may cause damage to the ocular surface before being neutralized by the tear fluid. This limitation was addressed by combining PAA with HPMC, a polymer that increases viscosity, resulting in the formation of pH-sensitive polymer mixtures that are a solution at pH 4 and a gel at pH 7.4. Blends of poly-methacrylic acid (PMA) and polyethylene glycol (PEG) have also been used as a pH-sensitive system to achieve gelation [40].

In this regard, Dawood and Kassab prepared and evaluated the behavior of pH-sensitive ocular naproxen gel in situ [41] to increase the residence time in the eye. These formulations were prepared using different concentrations of carbomer (0.5%, 0.6%, 0.7%) in combination with HPMC K40 (0.75%, 1%, 1.5%) or HPMC K100 (0.75%, 1%, 1.5%, 5%) [40]. The obtained gels were evaluated for appearance, pH, gelling capacity, tonicity, viscosity, in vitro release, and drug content, and subjected to release kinetic analysis, FT-IR studies, ocular studies, and irritability tests. The results showed that increasing the carbomer concentration improved both gelling capacity and gelling time. Furthermore, the higher the concentration of the hydrophilic HPMC polymer, the higher the viscosity of the formulation, thus affecting release, gelling ability, and time. Therefore, the formulation F10, made with CB 0.7% and HPMC K100 0.75%, showed an excellent in situ gel time activated by pH, and a sustained release of naproxen for 3 h with a release rate greater than 90%.

Wadetwar and collaborators investigated a new pH-sensitive in situ gel loaded with nanoparticles containing bimatoprost (BIM) for the treatment of glaucoma [42]. The nanoparticles loaded with BIM (BIM-SLN4) were then inserted into carbopol 941 to obtain nanoparticles loaded in the gelling system in situ (SLN-ISG). In particular, five batches (SLN-ISG1-SLN-ISG5) were prepared with a variation in the concentration of carbopol 941 from a minimum of 0.05% *w*/*v* to a maximum of 0.25%. SLN-ISG3 with 0.15 *w*/*v* was selected as the best batch due to its gelling capacity and viscosity. The formulations were in solution form at pH 5–6 and underwent sol-to-gel transformation when applied to eyes at pH 7.4. In vitro and ex vivo studies of BIM-SLN4 and SLN-ISG3 highlighted a prolonged drug release and its increased precorneal residence time. Following the HET-CAM test, it emerged that the formulation was not irritating and therefore well tolerated if applied to the eye. Histopathological studies also revealed no signs of tissue damage. Therefore, SLN-ISG may represent an interesting strategy for the treatment of glaucoma.

Kouchak et al. obtained a pH-triggered in situ gel system for ophthalmic delivery of dorzolamide (DRZ), a drug used for the treatment of ocular hypertension [43]. Different concentrations of carbopol and HPMC were used, but the optimal concentration for both polymers was found to be 0.1% because it had the character of a pseudoplastic fluid in both conditions. This carrier, according to the results of in vivo studies, showed better performance in retaining DRZ than the simple DRZ solution. Moreover, due to its high viscosity in situ and its mucoadhesive property, there was a prolongation of the precorneal residence time of the drug with consequent greater bioavailability, a decrease in the frequency of administration, and a reduction in systemic side effects. Therefore, this formulation can be a valid alternative to conventional DRZ eye drops [43].

Sheshala and coworkers developed and characterized in situ prolonged release ocular gels, containing sodium sulfacetamide, using pH-induced gelling polymers [44]. The formulations were obtained using carbopol 940/carbopol 934 alone or in combination with HPMC E4M. The appearance, pH, viscosity, gelling capacity, drug content, and in vitro drug release of these formulations were evaluated. The pH of all formulations was in the range of 5.9 to 6.7 and therefore compatible with the eye. Carbopol 940-based gels showed higher viscosity than carbopol 934 gels and drug release was also sustained for up to 8 h. The selected formulation containing 0.8% *w*/*v* of carbopol 940 (Loba Chemie Pvt. Ltd., India) and 1.5% *w*/*v* of HPMC E4M demonstrated similar antimicrobial efficacy to that of the commercial product. The most interesting advantage of these carbopol^®^/HPMC-based in situ gelling systems is their potential to improve patient compliance by reducing the dosing frequency of sodium sulfacetamide.

In 2019, Jain and collaborators investigated a pH-activated in situ gel useful for prolonged administration of levofloxacin in the dry eye treatment of eye infections [45]. For this purpose, HPMC, sodium alginate, and a boric acid buffer were used. The latter was chosen due to its antimicrobial activity. The formulation was presented in solution at pH 4.7 and rapidly turned into a gel with an increase to pH 7.4. The sustained release of levofloxacin from the in situ gel was observed for 24 h. In addition, the ex vivo permeation tests also showed a significant improvement in corneal permeation compared to conventional eye drops. Further studies on the antimicrobial efficacy revealed that the formulation is effective against *E. coli* and *S. aureus*, and the stability tests suggested that, due to its long period of stability, it can be considered a valid approach to the treatment of infected eyes.

#### 2.2.3. Ion-Sensitive In Situ Gel Systems

In the ions triggered in the in situ gelling systems, the viscosity of the solution increases with exposure to the ionic concentration of the lacrimal fluids. Ion-sensitive polymers can crosslink with the cations (monovalent and divalent) present in the tear fluid on the ocular surface and thus increase the residence time of the drug in the eye [46].

The first patented ion-sensitive in situ gelation system was Gelrite^®^ (DUCHEFA BIOCHEMIE B.V, RV Haarlem). The Netherlands, a low-acetyl gellan gum-based polysaccharide that forms transparent gels in the presence of mono or divalent cations [47]. The sodium concentration in the tears, 2.6 g/L, is particularly suitable for provoking gelling of the material when instilled locally in the conjunctival sac. Gelrite^®^ has been studied for the release of timolol, a beta-adrenergic blocking agent used locally in the reduction of elevated intraocular pressure. In vivo studies demonstrated that gel formation prolonged the precorneal residence time of timolol and increased its ocular bioavailability in the cornea, aqueous humor, and iris and ciliary body of albino rabbits. Subsequently, significant advances have been made and various materials have been investigated as ion-activated in situ gelling systems, such as gellan gum, alginates, deacetylated gellan gum, anionic polymers (carbopol), cationic polymers (chitosan), non-ionic polymers (HPMC, methylcellulose), polymers thiolates (thiomers), and carbomer.

In 2011, Rupenthal et al. compared several anionic polysaccharides (gellan gum, xanthan gum, carrageenan, and alginate) with an unfilled polymeric system (HPMC) and a positively charged polymeric system (chitosan), focusing on gelling behavior, rheological and textural properties, the microstructure of the gel, the contact angle, and the in vitro re-lease characteristics [48]. All systems appeared as physically intertwined polymer networks that favored the in vitro sustained release of a model hydrophilic drug versus a solution of the same drug. Although the formulations based on HPMC and chitosan did not undergo structural changes after the addition of cations, the formulations based on gellan gum and carrageenan showed a notable increase in viscosity, pseudoplasticity, and hardness following the addition of C^2+^ and K^+^ ions. This makes them interesting for ocular use because they can gel in contact with the cations of the tear fluid, with a noticeable lowering of the nasolacrimal drainage [48].

Subsequently, an ion-activated in situ gel ophthalmic delivery system was developed for ketotifen, an antihistamine drug, based on gellan deacetylase gum (DGG), and its rheological characteristics, stability, gelation, and release were investigated in vitro. In addition, the in vivo pharmacodynamic activity was also evaluated [49]. DGG is an extracellular polysaccharide, produced from *Pseudomonas elodea*, a Gram-negative bacterium separated from water lily [50] that features a parallel double helix structure, and the entire gel chain is composed of four base units for repeated curing. Therefore, when it is in solution at a certain concentration, it has the characteristic of gelling induced by cations. The DGG-based formulation exhibited optimal viscosity, which enabled easy administration in the form of drops, and which then underwent a rapid sol-gel transition due to ionic interaction. In vitro release showed that ketotifen release from in situ gels was moderate with no bursting effects. The formulation was almost stable over a storage period of 180 days. Furthermore, scintigraphic studies indicated that DGG is potentially useful in increasing the residence time of the formulation, with prolonged pharmacological effects compared to common drops.

Morsi et al. formulated an in situ gel based on ion-induced nanoemulsion for ocular administration of the antiglaucoma drug, acetazolamide (AZA), with the aim of obtaining a prolonged release of the drug and improved therapeutic efficacy [51]. Several formulations of acetazolamide-containing nanoemulsions have been prepared using peanut oil, tween 80, and/or chromophore EL as the surfactant, in addition to transcutol P or propylene glycol as solvent. Gellan gum at a concentration of 0.3% *w*/*w* showed optimal gelling when mixed with simulated tear fluid. The incorporation of xanthan gum, HPMC, and carbopol 940 did not exert a noticeable effect on in situ gelation, but greatly enhanced the mucoadhesive strength of gellan gum gel in situ. Based on the chemical-physical characteristics analyzed, the gellan/xanthan formulation showed good stability at all temperatures studied, and a longer intraocular pressure reduction effect than that of the marketed brinzolamide eye drops (Azopt^®^- Novartis Pharmaceuticals Canada Inc. 385 Bouchard Blvd.) and oral acetazolamide tablets (Cidamex^®^-Chemical Industries Development Building Street: Othman Moharram Street 2 Area: Pyramids Area P.O. Box: 12111 Country: Egypt City: Giza). This formulation can be considered to be a promising dosage form because, when applied topically for the treatment of glaucoma, it reduces the systemic side effects of AZA. It is also able to exert a prolonged therapeutic effect, which would improve patient compliance due to the reduced frequency of administrations [51].

In 2017, Kotreka and collaborators prepared in situ gel-forming eye drops based on estradiol activated by ions, which are useful in preventing the onset of cataracts in old age [52]. The eye drops were then formulated with gellan gum as an ion-activated gelling polymer, polysorbate-80 to promote drug solubility, mannitol to adjust tonicity, and potassium sorbate and disodium edetate dihydrate (EDTA) as preservative agents. The solution eye drops gelled when mixed with simulated tear fluid and gel formation was confirmed by viscoelastic measurements. A share of 80% of the drug from the gel was released in 8 h. The formulations were clear, isotonic with an adequate pH and viscoelastic behavior, and stable under accelerated and long-term storage conditions for 6 months. Therefore, the obtained results suggest the possibility that the formulation developed based on estradiol may be potentially suitable in preventing and delaying cataracts.

In recent work, Nair and collaborators designed, formulated, and evaluated the performance of an ionic gel in situ to improve ocular penetration and therapeutic activity of moxifloxacin [53]. The latter is a fluoroquinolone-lone derivative with a significant activity against many Gram-negative and Gram-positive pathogens. The formulation was prepared with polymers such as gellan gum, sodium alginate, and hydroxypropyl methylcellulose. The selected formulation, containing the above three polymers in equal parts, was evaluated by ex vivo permeation, in vivo irritation, and pharmacokinetics parameters in rabbits. The obtained results showed an increase in the concentration of polymers, and a rise in gel and adhesive strength and viscosity, but a decrease in the amount of drug. The formulation, however, maintained all physicochemical properties within acceptable limits, remained stable for 6 months, and was safe and non-irritating to the eyes. Significant improvement in moxifloxacin was observed compared to commercial eye drops. These results indicate that the developed in situ gelling system may offer more effective and extensive ophthalmic therapy than moxifloxacin in eye infections compared to conventional eye drops.

### 2.3. Intravitreal Injection

**Figure 4:**
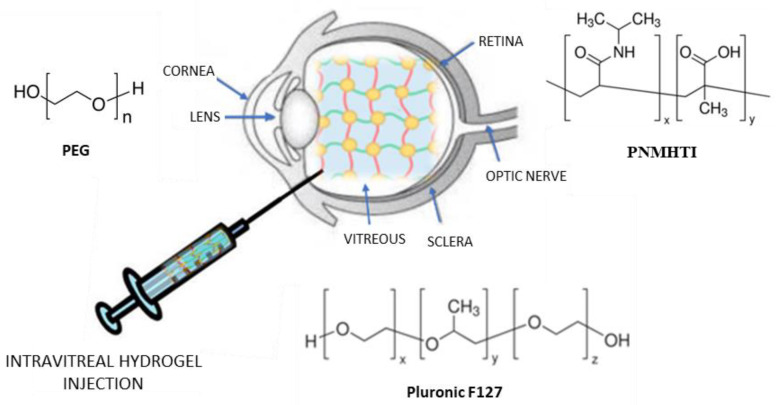
Hydrogel intravitreal administration.

The intravitreal administration of drugs is a strategy that allows high drug concentrations in the vitreous, thus avoiding adverse effects deriving from systemic administration. Unfortunately, repeated administrations are necessary to enable drugs to maintain a therapeutically effective concentration, which can cause damage to the lens and detachment of the retina [54]. For this reason, new delivery systems have been developed, including hydrogels, to improve the intravitreal delivery of drugs (Figure 4) and support the release of therapeutic proteins [55].

In this regard, Pachi et al. developed new hybrid drug-in-liposome-in-hydrogel formulations to prolong the retention, or support the release, of non-steroidal anti-inflammatory drugs (NSAIDs), such as Flurbiprofen (FLB), after intravitreal injection [56]. This system significantly improves the levels and duration of FLB in ocular tissues, demonstrating the role and biocompatibility of Pluronic F127 as a component of these formulations. This polymer is also sensitive to heat (liquid at low temperature and gel at body temperature); therefore, these hybrid formulations are easier to inject. Furthermore, due to the presence in the hydrogel of nanometric liposomes, translucent formulations have been obtained, which prevent the inconvenience of blurred vision.

More recently, Sapino and colleagues designed ophthalmic delivery systems of nanocomposites based on in situ gelling formulation injected into the vitreous cavity, to prolong (up to one week) the effect of cefuroxime (CEF) in the treatment of endophthalmitis, a severe intravitreal inflammatory disease [56]. For this purpose, two different nanocomposite systems, based on solid lipid nanoparticles (SLNs) or nanoemulsions (NEs), thickened with Pluronic^®^ F127 (Sigma Aldrich Co., St. Louis, MO, USA), were developed and their ability to slow the release of the trapped drug was evaluated. In particular, the formulations were prepared by adding the pluronic to the SLN and µE dispersions, both loaded with dCEF that was prepared by esterifying CEF with 1-dodecanol, to obtain a lipophilic CEF derivative to be trapped in the SLN lipid matrix or dissolved in the µE. These formulations were evaluated for their physical appearance, content of the drug, temperature of the gelification, and rheological properties of injectability. The authors also performed in vitro release studies and stability tests. Furthermore, cell proliferation assays on epithelial cells of the human retinal pigment ARPE–19 were performed to evaluate the influence of this innovative system on cell viability. The preliminary data presented by Sapino et al. on nanocomposite hydrogels can be considered to be a starting point for the intravitreal administration of drugs useful in age-related degenerative diseases of the retina [57].

In 2019, Zou et al. produced an in situ thermosensitive injectable hydrogel conjugated with indomethacin to avoid repeated administration of this drug in the treatment of chronic ophthalmic diseases such as uveitis [58]. Poly (NIPAAmco-MAA-co-HTI) (PNMHTI) hydrogel, based on N-isopropylacrylamide (NIPAAm), methacrylic acid (MAA), and 2-hydroxyethyl methacrylate-g-poly (tri-methylene carbonate)-indomethacin (HEMA-g-PTMC-IND) (HTI), was obtained by radical polymerization of NIPAAm, MAA, and HTI. Rheological tests showed the solution is gelled (sol-gel transition) at a physiological temperature of 37 °C. The release of indo-methacin from injectable hydrogels was prolonged for more than 2 weeks. Furthermore, the hydrogels showed excellent biocompatibility towards mouse fibroblasts, reduced in-domethacin cytotoxicity, and showed a high anti-inflammatory effect against macrophages. Uveitis was treated satisfactorily in New Zealand rabbits. Therefore, PNMHTI hydrogels represent an important strategy in the treatment of chronic ophthalmic diseases.

In 2018, Anwary et al. presented a review of various biodegradable and non-biodegradable injectable intravitreal polymeric hydrogel devices, such as poly (ethylene glycol), poly (lactic-co-glycolic acid), silica, hyaluronic acid/dextran, silk, chitosan/alginate, and polymers based on (N-isopropylacrylamide). In particular, the authors described their hydrogel compositions, in addition to the effects resulting from their use. Therapeutic agents administered in these devices were also described, such as the monoclonal antibody ranibizumab, the corticosteroid drug dexamethasone, Avastin^®^/bevacizumab, and the anticancer drug Aflibercept. The indisputable importance of using the aforementioned intelligent polymeric systems for drug delivery to the posterior segment of the eye was therefore emphasized [59].

## 3. Contact Lenses

Contact lenses are ocular prosthetic devices that have several functions, such as the correction of refractive errors in the cases of myopia, hypermetropia, and astigmatism [60]. These devices are used to treat ocular dysfunctions, particularly corneal irregularities, and for post-surgical refractive rehabilitation. However, they can also be used as cosmetic lenses, such as colored and limbal ring lenses. Another interesting application of contact lenses concerns the prolonged administration of drugs [61] first described by Sedlacek in 1965 [62]. Subsequently, significant attention has been paid to the ability of contact lenses to improve corneal penetration and drug bioavailability [63,64]. The lens absorbs some of the drug from the tear film and then acts as a reservoir, slowly releasing the drug into the tears as the overall drug concentration in the tear film decreases. For this purpose, two methods are used. The lenses can be immersed in a solution of the drug for a period of time and then placed on the eye, resulting in a high initial release, followed by a slower and long-term release during the hours following the application, as in the administration of antibiotics or non-steroidal anti-inflammatory drugs. Alternatively, the drug can be applied to the contact lens after its application in the eye. This method is often adopted when the lens acts as a protective device (bandage lens), for example, following a corneal injury [65]. However, both approaches prolong the contact time of the drug by improving its penetration through the cornea [66].

**Figure 5:**
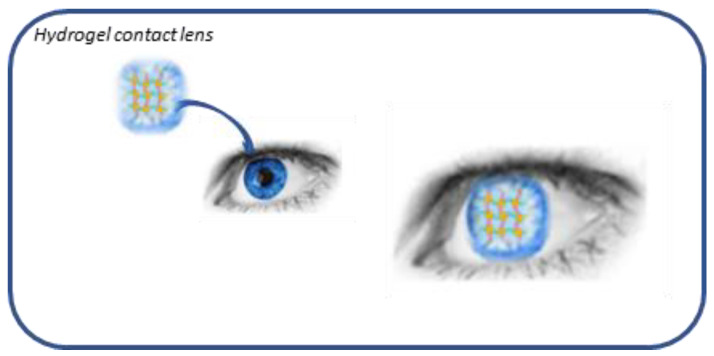
Hydrogel-based contact lenses.

The materials used for the manufacture of contact lenses include hydrogels (Figure 5). Because hydrogels are composed of hydrophilic monomers containing electrochemical polarities, they can allow interaction with water. In addition, they are also oxygen permeable and flexible, and capable of retaining a large percentage of water within their polymer network. Due to these characteristics, hydrogels are an attractive material for the production of contact lenses. [67,68]

However, traditional hydrogels do not show excellent properties in encapsulating and controlling drug release due to the simple hydrophilic polymer chain, which does not undergo any additional interaction with the drug molecule. For this reason, Hu and collaborators synthesized functional hydrogels that are useful for the administration of ophthalmic drugs in the therapy of oculopathy [69]. Specifically, the functional monoglucose methacrylate monomer (GMA)-β-cyclodextrin (mono-GMA-β-CD) and the functional methacrylate-β-cyclodextrin crosslinker (MA-β-C) were incorporated into the hydrogel by copolymerization in such a ratio to condition the swelling at equilibrium, the contact angle, the viscoelasticity, and the surface morphology of the hydrogel. Furthermore, the functional hydrogel containing the β-CD domain showed a better resistance to proteins and a significantly greater amount of encapsulated drug at equilibrium compared to the traditional hydrogel. Furthermore, in vivo studies have highlighted the better performance of functional hydrogels in reducing intraocular tension even compared to commercial eye drops.

In 2017, Horne et al. proposed an interesting non-aqueous approach for loading hydrophobic drugs into silicone hydrogel contact lenses and, in this regard, latanoprost was chosen as the model hydrophobic drug [70]. Then, by immersing the lens in a n-propanol solution of the drug, the lens absorbed, in about 4 min, the organic solvent, causing the active ingredient to be dissolved into the contact lens by convection of the solvent, rather than by diffusion. Furthermore, it was shown that, through this loading system, the amount of hydrophobic drug inserted into the lenses was controllable, up to 450 μg per lens, and was proportional to the loading time and the concentration of the drug in the solution. The in vitro release of the drug from the lenses into the simulated tear fluid was proportional to the total amount of drug loaded, and diffusion was controlled for the first 3 days and completed within 4 days. The important result of this study was the demonstration of how loading a hydrophobic drug into silicone hydrogel contact lenses can occur within minutes and provide sustained release for many days.

In recent work, Silva and collaborators created a silicone-based hydrogel with a molecular imprint to be used in the production of contact lenses capable of releasing the antibiotic moxifloxacin hydrochloride (MXF) at an adequate rate for the treatment of eye infections (5 to 7 days), which is the recommended duration for topical treatment with conventional eye drops [71]. Hydrogels were then prepared by the molecular imprinting method using acrylic acid (AA) as a functional monomer for the specific recognition of the antibiotic. In vitro experiments mimicking ocular surface fluid turnover showed that the imprinted hydrogel with the highest AA content released the MXF in the release medium at a concentration effective against *S. aureus* and *S. epidermidis* for approximately 2 weeks. Typical parameters of contact lenses were also analyzed, such as water absorption, wettability, transmittance, ionic permeability, and Young’s modulus. It was found that, even in the modified hydrogel, these parameters remained within the recommended ranges of values for contact lenses. Furthermore, the developed contact lenses did not show cytotoxicity and ocular irritation effects. Therefore, they may represent a promising strategy for the treatment of ocular infections.

## 4. Conclusions

Eye drop solutions are the most widely used ophthalmic pharmaceutical forms for the treatment of eye diseases of the anterior segment. However, they have many limitations, including low bioavailability, the need for high doses, and poor patient compliance. Due to these reasons, in recent years, pharmaceutical research has focused attention on the development of new drug administration approaches aimed at increasing the ocular residence time, to provide a prolonged pharmacological action, thus improving both bioavailability and patient safety, and therefore minimizing side effects. Among the related implemented strategies, hydrogels represent an ideal delivery system because they are an extremely versatile class of materials, biocompatible with many potential applications in ophthalmology. They can be used as gel eye drops, as in situ gelling formulations, in intravitreal injections, or as therapeutic contact lenses (Table 1). In this context, this review aimed to analyze gel-based materials and their main applications in ophthalmology. The works described in this paper demonstrate the unquestionable advantages deriving from the use of hydrogels to increase the residence time of drugs in the precorneal area and thus improve their bioavailability.

## Figures and Tables

**Table 1:** Gel-based formulations.

Gel-Based Formulations	Type of Polymer	Origin	Behaviour	References
***Gel eye drops*** 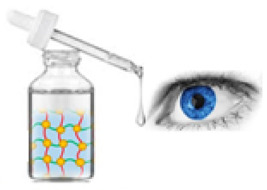	*Carboxymethylcellulose (CMC)*	*Semi syntetic*	***Viscous solutions before administration into the eye***	[12,13]
	*Hydroxypropylmethylcellulose (HPMC)*	*Semi syntetic*		[14,15]
	*Carbomer (carbopol)*	*Synthetic*		[16]
	*Hyaluronic acid (HA)*	*Natural*		[17,18]
	*Alginic acid*	*Natural*		[19]
	*Chitosan*	*Natural*		[23]
***In situ gels*** 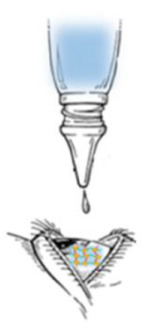	*Poly (N-isopropylacrylamide)hyaluronic acid (PN-HA)*	*Synthetic/* *Semi syntetic*	***Temperature-sensitive*** ***in situ gel systems***	[34,39]
	*Poloxamer/ Carboxymethylcellulose (CMC)*	*Synthetic/* *Semi syntetic*		[35,37]
	*Hydroxypropylmethylcellulose* *(HPMC)*	*Synthetic/* *Semi syntetic*		[36,45]
	*Carbomer/Hydroxypropylmethylcellulose* *(HPMC)*	*Synthetic/* *Semi syntetic*	***pH-sensitive*** ***in situ gel systems***	[41]
	*Carboplol*	*Synthetic*		[42,43,44]
	*Gellan gum, xanthan gum, carrageenan, and alginic acid*	*Natural*	***Ion-sensitive*** ***in situ gel systems***	[48,49,50,51,52,53]
***Intravitreal injection*** 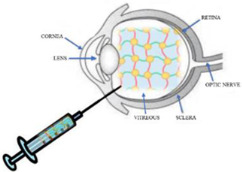	*Pluronic F127*	*Synthetic*	***Gel injected into the vitreous cavity***	[56,57]
	*Poly (NIPAAm-co-MAA-co-HTI) (PNMHTI)*	*Synthetic*		[58]
***Contact lens*** * 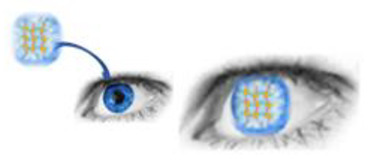 *	*Monoglucose methacrylate monomer*	*Synthetic*	***Ocular prosthetic devices***	[69]
	*Silicone*	*Synthetic*		[70]
	*Acrilic acid*	*Synthetic*		[71]
